# Ensemble of One-Class Classifiers for Personal Risk Detection Based on Wearable Sensor Data

**DOI:** 10.3390/s16101619

**Published:** 2016-09-29

**Authors:** Jorge Rodríguez, Ari Y. Barrera-Animas, Luis A. Trejo, Miguel Angel Medina-Pérez, Raúl Monroy

**Affiliations:** Escuela de Ingeniería y Ciencias, Tecnologico de Monterrey, Carretera al Lago de Guadalupe Km. 3.5, Atizapán, Edo. de México C.P. 52926, Mexico; jorger@itesm.mx (J.R.); A01373306@itesm.mx (A.Y.B.-A.); migue@itesm.mx (M.A.M.-P.); raulm@itesm.mx (R.M.)

**Keywords:** behavior analysis, classifier ensemble, personal risk detection, one-class classification, wearable sensor

## Abstract

This study introduces the One-Class K-means with Randomly-projected features Algorithm (OCKRA). OCKRA is an ensemble of one-class classifiers built over multiple projections of a dataset according to random feature subsets. Algorithms found in the literature spread over a wide range of applications where ensembles of one-class classifiers have been satisfactorily applied; however, none is oriented to the area under our study: personal risk detection. OCKRA has been designed with the aim of improving the detection performance in the problem posed by the Personal RIsk DEtection(PRIDE) dataset. PRIDE was built based on 23 test subjects, where the data for each user were captured using a set of sensors embedded in a wearable band. The performance of OCKRA was compared against support vector machine and three versions of the Parzen window classifier. On average, experimental results show that OCKRA outperformed the other classifiers for at least 0.53% of the area under the curve (AUC). In addition, OCKRA achieved an AUC above 90% for more than 57% of the users.

## 1. Introduction

Personal risk detection [1] has been defined as the timely identification of when someone is in a dangerous situation, such as a health crisis or car accident or other events that may endanger a person’s physical integrity. To structure this problem, Barrera-Animas et al. [1] suggested that people usually behave according to the same behavioral and physiological patterns or small variations of them. A risk-prone situation should produce sudden and significant deviations in these user patterns, and the changes can be captured by a group of sensors, such as an accelerometer, gyroscope and heart rate monitor, which are normally found in current wearable devices. The problem posed in [1] is an anomaly detection problem, where the aim is to distinguish an unusual condition (possibly a risk-prone situation) from a normal behavior. One-class classification mechanisms have proven effective in this context. Indeed, Barrera-Animas et al. reported in [1] that a one-class support vector machine (ocSVM) achieved the best performance and best average ranking compared with other classifiers in tests.

Techniques for combining one-class classifiers aim to improve the classification performance provided by single classifiers. Additionally, a promising approach for enhancing an ensemble involves training one or several classifiers of the ensemble with different features of the dataset, where the most common techniques are random feature selection methods, such as the random subspace method (RSM, which is also called attribute bagging) and random partitions, subspace clustering, projection methods, bagging and rotation forest [2,3,4,5,6,7,8,9,10,11,12,13].

In this regard, Tax and Duin [2] and Nanni [4] used feature selection in order to train every single classifier in an ensemble. The results obtained in both studies demonstrated that combining features is more effective than combining different classifiers and, in general, produces better results than the individual classifier counterpart.

Juszczak and Duin [3], Biggio et al. [5] and Medina-Pérez et al. [13] aimed to improve the accuracy of classification by employing ensembles based on several instances of the same base classifiers. The techniques used in [3,5] for feature subspace partition included fixed combining rules, RSM and bagging. Juszczak and Duin successfully minimized the number of classifiers considered for the ensemble, whereas Biggio et al. and Medina-Pérez et al. obtained improvements in both the classification accuracy and robustness when they compared their ensemble approaches with state-of-the-art classifiers.

Alternatively, Cheplygina and Tax [6] proposed applying pruning to an RSM of one-class classifiers; they demonstrated that the performance could be noisy using RSM, and pruning inaccurate classifiers from the ensemble was more effective than using all of the available classifiers. Recently, Krawczyk in [8] proposed a technique for producing efficient one-class classifier ensembles by combining a pruning algorithm with a weighted fusion module that controls the influence of the selected classifiers on the final ensemble decision. The experimental results demonstrated that in most cases, this method outperformed the state-of-the-art pruning algorithms for selecting one-class classifiers from an ensemble.

Therefore, one-class classifier ensembles combined with classifier pruning and random feature selection have the ability to outperform state-of-the-art one-class single classifiers in most cases. In general, the ensembles exhibit robustness and diversity, which allow them to obtain better classification accuracy.

Table 1 presents a summary and a brief comparison of the methods used by the studies reviewed in this work. As can be noted, a wide range of applications has been considered, where ensembles of one-class classifiers were applied satisfactorily, but none were used in our area under study: personal risk detection.

The aim of this study is to improve the accuracy of the detection results reported by Barrera-Animas et al. [1]. To accomplish this, a new algorithm is proposed and its applicability to the personal risk detection problem demonstrated. The proposed algorithm called One-Class K-means with Randomly-projected features Algorithm (OCKRA) is an ensemble of one-class classifiers, built over multiple projections of the dataset according to random subsets of features. OCKRA comprises 100 classifiers, each of which is built upon 10 centers computed by k-means++ [14] with Euclidean distance. In the training phase, each individual classifier applies k-means [15] to a random projection of the dataset and stores the centroids of the clusters. In the classification phase, to determine whether a query object is a risk-prone situation or a normal behavior, each classifier compares it with all of the centroids in order to determine the cluster to which the object is most likely to belong. Each classifier returns a similarity value according to the distance of the query object relative to its closest cluster centroid. The ensemble returns the average similarity computed by individual classifiers.

To evaluate the performance of OCKRA and to compare it with other classifiers, the Personal RIsk DEtection (PRIDE) dataset described in [1] was used. PRIDE is built based on 23 test subjects where the user data were collected in ordinary life activities, as well as specific scenarios under stressful conditions. Data were captured for the users by employing a set of sensors embedded in a wearable band. The classifiers were trained based only on the daily behavior of the users. Then, the performance of the classifiers was tested while detecting anomalies that were not included in the training process for the classifiers.

OCKRA was compared with the following classifiers: ocSVM [16], the Parzen window classifier using Euclidean distance [17] and two versions of the Parzen window classifier based on k-means. In the first version, k-means classifies new objects based only on the closest center of the cluster [2], whereas in the second, k-means classifies new objects using all of the centers of the clusters [18]. On average, results showed that OCKRA outperformed the other algorithms for at least 0.53% of the area under the curve (AUC). This number is small, but it is significant because a user will have a higher probability of being assisted in time if they encounter a risk-prone situation. Experimental results are encouraging because the classifier achieved an AUC greater than 90% for more than 57% of the users.

The main contribution of the present study is a new algorithm based on an ensemble designed for one-class classification, which is suitable for personal risk detection. During the design process, it was necessary to consider that OCKRA should run on a mobile personal device, such as a smartphone, with limited resources in terms of the CPU and memory. These restrictions affect the ensemble parameters, i.e., a small value for *k* (the number of clusters) and the number of classifier instances, while maintaining good classification accuracy. In addition, the final design had to be capable of efficiently integrating any new learning object into the ensemble’s knowledge base without the need to retrain every single classifier.

Before introducing the new algorithm in Section 3, the dataset used for the experiments is described in the following.

## 2. PRIDE Dataset

The PRIDE dataset was described in [1], and it is based on ordinary daily activities as well as specific scenarios under stressful situations. The PRIDE dataset was built based on 23 test subjects during a data collection period of one week for 24 h each day to obtain the normal conditions dataset (NCDS). A Microsoft Band v1^©^ and a mobile application developed by the authors using the available SDK were used to collect the data. All of the captured stream data were transferred to a private cloud under our control.

Next, to build the anomaly conditions dataset (ACDS), the same 23 test subjects participated in another process to acquire data under particular conditions, where five scenarios to simulate abnormal or stressful conditions were designed, which included the following activities: rushing 100 m as fast as possible, going up and down the stairs in a multi-floor building as fast as possible, a two-minute boxing practice session, falling back and forth and holding one’s breath for as long as possible. Each activity aimed to simulate a dangerous or abnormal condition in the real world, e.g., running away from a dangerous situation, leaving a building due to an evacuation alert, fighting an aggressor during a quarrel, swooning and experiencing breathing problems, such as dyspnea. The session required building the anomaly dataset for each test subject lasting for about two hours, and it demanded major physical effort.

In order to obtain reliable data that could help to distinguish abnormal from normal conditions, the daily collection of behavior and vital signs data was not restricted to a laboratory environment; on the contrary, test subjects felt comfortable wearing the band [19], and they were not deprived of their privacy [20]. Furthermore, the ACDS data collection process was monitored without interfering with the test subjects in terms of their freedom to perform activities.

The daily log obtained from a test subject included all of the observations that occurred from 00:00 h to 23:59 h. Data were collected for a test subject during a period of seven days, but there were gaps in the log of at least 40 min usually for three times per day due to the battery recharging process. Moreover, the data were collected during ordinary daily tasks, so the test subjects usually paused the data collection process for personal reasons on several occasions, e.g., to perform an aquatic activity.

To perform a more comprehensive study [21], test subjects with diverse characteristics in terms of gender, age, height and sedentary lifestyle were considered. The test subjects comprised eight female and 15 male volunteers aged between 21 and 52 years, with heights from 1.56 to 1.86 m, weights from 42 to 101 kg, exercising rates of 0 to 10 h a week and the time spent sitting during working hours or leisure ranging from 20 to 84 h a week. The elderly comprise a very important group in society, but were not included in the data collection process due to the demanding nature of the method employed.

PRIDE is freely available for downloading through the Syncplicity^©^cloud solution by sending a request to the corresponding author. For the interested reader, Annex 1 shows the mean value, $\bar{x}$, and the sample standard deviation, *s*, for all of the test subject features, as well as the number of observations from the PRIDE dataset. All of the procedures performed in this study involving human participants were conducted in accordance with the national law of the Protection of Personal Data in Possession of Particulars. Informed consent was obtained from all of the individual participants in the study.

Next, a brief description of the mobile application developed, as well as the band’s built-in sensors is given.

### 2.1. Mobile Application and Sensor Network Description

To capture data from the test subjects, a Microsoft Band [22,23] was used because of two main reasons: the number and nature of sensors included in the band; and the availability of an SDK that allowed us to develop an application according to our needs. The application was developed for the Android platform, and it was connected to the band via Bluetooth. The application acquired the sensor data in real time and stored all of the measurements in a CSV file on the mobile phone. The file was transferred to a secure FTP server under our control, which was performed by the test subject once or twice each day by using the upload file to server option on the application.

Accelerometer and gyroscope data were acquired at an interval of 125 ms (the sensor operating frequency was set to 8 Hz) in order to maintain a reasonable file size (about 175 MB) after 24 h and a battery life of almost 9 h. The battery could be recharged to 80% of its full capacity within approximately 40 min. For comparison, if a sensor operating frequency of 31 Hz or 62 Hz were used, the size of the file would have increased to 650 MB and 870 MB, respectively, and the battery life would have degraded to 8 h and 6 h, respectively. However, the frequency of operation could be set directly to 8, 31 or 62 Hz at any time from the application. Distance, heart rate, pedometer and calorie measurements were logged using a readout interval of 1 s, and UV and skin temperature data were collected every 60 s and 30 s, respectively, or whenever the value changed. GPS data were not available using the SDK, so it should be noted that the distance was derived using a proprietary Microsoft algorithm, which considered the number of steps taken by the user (pedometer), the user’s stride length and their height. Height and other types of general user information were provided when setting up the application and during synchronization with the band for the first time. The algorithm was patented by Microsoft (Adaptive Lifestyle Metric Estimation. Microsoft Internal Number 341468. U.S. Patent 20150345985-A1).

Table 2 describes the sensors in the band, as well as the frequencies at which their data could be retrieved. Using the sensor operating frequencies shown in the table, the test subject data could reach approximately 1,670,160 records per day (around 175 MB), and the battery life was about 9 h.

### 2.2. PRIDE Pre-Processing for Online Personal Risk Detection

Before using PRIDE and performing any experiments, the data had to be prepared in order to derive a feature vector for every test subject, which was refreshed every second. The following steps summarize the dataset pre-processing procedure.

- For each test subject in the PRIDE dataset, the records were arranged by day.
- The feature vector was computed using a window size of one second. The frequency of our sensors varied significantly, so three rules were applied to compute the feature vector for a given window: (1) if the readout interval was less than one second, the feature vector was assigned both the average and sample standard deviation for all of the sensor measurements; (2) if it was equal to one second, then the feature vector was assigned directly the sensor value; and (3) if it was greater than one second, the feature vector was assigned the last sensor value.

Specifically, a feature vector for a given window contained the following:

- Means and standard deviations for the gyroscope and accelerometer measurements;
- Absolute values obtained by the heart rate, skin temperature, pace, speed and UV sensors;
- The incremental changes (Δ-value) in the absolute values for the total steps, total distance and calories burnt; a Δ-value was computed as the difference between the current and previous values.

Thus, a 26-dimensional feature vector was obtained, and its values were refreshed every second. The structure of this vector is shown in Table 3 and Table 4. Both the NCDS and ACDS were pre-processed using this method.

Next, each of the test subject logs was divided into five folds to use them in a five-fold cross-validation. In the cross-validation, four folds of the normal behavior by test subjects were used for training, and one fold was joined with the anomaly dataset log to test the classifiers. This procedure was repeated five times by alternating the test subject fold that was retained for testing. Thus, five training datasets and five testing datasets were obtained.

In our datasets (the pre-processed PRIDE dataset), every object was stored as a row in a CSV file, which followed the structure of the feature vector described previously. The last column of every row contained one of two labels: “typical” or “atypical”. The label “typical” indicated that the object represented normal test subject behavior, and the label “atypical” indicated that the object represented an anomalous state. This label was never used for training the classifier, and it was only used for testing purposes.

Next, the new algorithm will be described in detail as an ensemble of one-class classifiers for personal risk detection.

## 3. Proposed Algorithm

This study introduces a new algorithm called OCKRA, which is based on the hypothesis that a risk-prone situation produces sudden and significant deviations from standard user patterns. These patterns are computed based on the data sensed by the Microsoft Band, as described in Section 2.1.

The proposed algorithm is an ensemble of one-class classifiers, based on multiple projections of the dataset according to random subsets of features. Random subsets of features are used to ensure that there is high diversity [6,7,8] among the classifiers, in the same manner as some other well-known ensembles, e.g., random forest [24]. OCKRA first applies k-means++ [14] to each subset of features to obtain a collection of cluster centroids. Next, to classify a new sample observation, OCKRA returns an average similarity measurement, which is computed by the ensemble of all of the individual classifiers. While combining multiple k-means, each one computed on a random projection of the dataset, has already been explored for clustering [15], the novel aspect of our method is that OCKRA is designed for one-class classification problems instead of several-class problems. In particular, OCKRA operates in the context of personal risk detection based on wearable sensors, where processing is performed by devices with limited resources, such as smartphones. In the following, a formal description of the ensemble training and classification phases is given.

### 3.1. Training OCKRA

OCKRA is an ensemble of one-class classifiers, each of which is based on k-means++ (Algorithm 1). As described by Breiman in [24], the number of individual classifiers was set at 100, which yielded good experimental results, but determining the best number of classifiers with respect to detection performance remains an open question. In order to introduce diversity among each of the elements of the ensemble, a different set of features was used in order to train each individual classifier. To train the ensemble, the process starts with an initial training dataset *T* of size m×n, where *m* is the number of samples and *n* is the number of features. Random feature selection with replacement was used (Step 4). In this step, *n* random numbers between one and *n* are generated using a uniform probability distribution. After removing any duplicates, the remaining elements represent the index of the features that will be considered during the construction of individual classifiers. According to our experience, this procedure extracts 63% of the *n* original features on average.

Next, for each classifier, the algorithm projects the training dataset *T* (Step 5) over the randomly-selected features in order to obtain the projected dataset $T^{\prime }$. This dataset has a size of $m\times n^{\prime }$, where $1\leq n^{\prime }\leq n$ is the number of randomly-selected features. Next, the algorithm computes the centers of the *k* clusters obtained using k-means++ (Step 7). The distance function is set to Euclidean (which is standard in previous studies) and *k* = 10. *k* needs to be small since OCKRA must work online using smartphones, so it should consume low RAM memory and CPU resources while maintaining good classification accuracy. Again, determining the optimum value of *k* with respect to the consumption of resources and detection performance remains an open question. The algorithm uses the centers of the clusters obtained by k-means++ to build one-class classifiers, which return the likelihood that a new object belongs to its nearest cluster given a distance threshold. To compute the distance threshold (Step 6) and because of the massive amount of available data for each user, the process uses a subset of $T^{\prime }$ created by using only the data points separated by 60 s between them, which reduces the data required for processing from *m* samples to approximately m/60. The distance threshold is computed by averaging the distance between all of these points. It was decided to use the average distance after experimenting with different thresholds, such as the maximum and minimum distance between points, because it performed better in different contexts.

The training phase returns a set comprising the parameters of each individual classifier, which consists of a triplet containing the randomly-selected features, the computed centroids of each cluster and the distance threshold.

**Algorithm 1** OCKRA training phase.  1:**function** OCKRA_Train(*T*)                ▷*T*: training dataset.  2:    OCKRAParameters←{}  3:    **for** i=1..100 **do**  4:        SelectedFeatures← RandomFeatures(T)  5:        $T^{\prime }\leftarrow$ Project(T,SelectedFeatures)  6:        $\delta_{i}\leftarrow$ AvgDistances$\left(T^{\prime }\right)$  7:        Centres← ApplyKMeansAndComputeCentres$\left(T^{\prime }\right)$  8:        $OCKRAParameters\leftarrow OCKRAParameters⋃\{\left(SelectedFeatures,Centres,\delta_{i}\right)\}$  9:    **end** **for**10:    **return** OCKRAParameters11:**end** **function**


### 3.2. Classification Using OCKRA

In order to classify a new object, the algorithm takes a one-dimensional table *O* containing the object, which is a vector of size 1×n (Algorithm 2). The following steps need to be performed for each classifier parameter triplet obtained from the training phase. First, OCKRA projects *O* onto the randomly-selected feature space (Step 5) obtained by training, thereby resulting in a projected object contained in $O^{\prime }$. After projection, the first step for classification by OCKRA involves selecting the nearest cluster to $O^{\prime }$, which is achieved by selecting the centroid with the smallest Euclidean distance to the object in $O^{\prime }$ (Step 6).

The last step of classification with OCKRA involves transforming the distance between the projected object and its nearest cluster centroid onto a similarity value in the interval [0,1] (Step 7). A value of zero indicates a risk-prone behavior, whereas a value of one indicates that the object resembles the normal behavior of users. If the distance value is less than $\delta_{i}$, the algorithm computes a high similarity value (>0.6), which indicates that the object is likely to belong to the cluster of its nearest center. When the distance is more than three times $\delta_{i}$, the algorithm yields a low similarity value (<0.02), which indicates that the object does not belong to any cluster. It is worth noting that there is an inverse relationship between the similarity score and the distance from the object to the nearest cluster.

In order to reach a consensus among all of the members of the ensemble, OCKRA averages the similarity measurements of all of its classifiers (Step 9).

The ensemble returns a similarity value where zero indicates a potentially risky situation and one represents normal behavior, so the accuracy of our classifier relies on a threshold for determining what is anomalous and what is not. A threshold value closer to one will detect most of the risk-prone situations, but it may annoy the user by confusing normal behavior with risk. By contrast, if the threshold is closer to zero, the user would be notified less often, but the system is likely to miss many actual risky situations. Hence, the setting of the threshold value depends on the specific needs of the user (e.g., detecting risky situations for a healthy athlete is not the same as that for an elderly person in a retirement home).

**Algorithm 2** OCKRA classification phase.  1:**function** OCKRA_Classify(O,OCKRAParameters)  2:    ▷*O*: table that contains object to be classified; *OCKRAParameters*: set returned by Algorithm 1  3:    s←0  4:    **for** **each** $(Features_{i},Centres_{i},\delta_{i})\in OCKRAParameters$ **do**  5:        $O^{\prime }\leftarrow$ Project$\left(O,Features_{i}\right)$  6:        $d_{min}\leftarrow \min_{c_{j}\in Centres_{i}}($EuclideanDistance$\left(O^{\prime },c_{j})\right)$    7:        $s\leftarrow s+e^{-0.5{\left(d_{min}/\delta_{i}\right)}^{2}}$  8:    **end** **for**  9:    s←s/|OCKRAParameters|10:    **return** *s*11:**end** **function**


## 4. Results and Discussion

By using the method described in [1], the performance of OCKRA was tested based on a five-fold cross-validation, as described in Section 2.2, during the pre-processing step with the PRIDE dataset.

Users want to be protected by an ideal classifier, which can correctly discriminate every possible abnormal behavior from that of a normal user. Therefore, the aim is to build classifiers that maximize true positive classifications (i.e., true abnormal conditions) while minimizing false positive ones (i.e., false abnormal or dangerous situations). Therefore, the classifiers were evaluated using the following performance indicators.

- Precision-recall (P-R) curves: Precision refers to the fraction of retrieved instances that are relevant, and recall (also known as sensitivity) is the fraction of relevant instances that are retrieved. In our case, a relevant instance is characterized by a true anomaly. In addition, recall is equivalent to the true positive detection rate (TPR). A P-R curve was built for each user independently, as well as a single P-R curve based on the mean and standard deviation for all of the users.
- ROC curves: The performance indicators were computed based on the receiver operating characteristic (ROC) curves, built according to Fawcett [25]. A similar approach to that used for constructing the P-R curves was employed. A ROC curve was built for each user independently and also a single ROC curve based on the mean and standard deviation of the total population. Sensitivity is crucial in a personal risk detection context, where it is preferable to receive several false alerts (false abnormal or dangerous situation), rather than missing one true one (false ordinary or normal condition); hence, it is important to maximize recall even at the cost of experimenting with a certain false alarm rate.
- AUC: The AUC of the TPR versus the false positive detection rate (FPR), which indicates the general performance of the classifier for all FPR rates.

OCKRA was compared with the following classifiers.

- ocSVM: The implementation of ocSVM [16] included in LibSVM [26] with the default parameter values (γ=0.038 and ν=0.5) and using the radial basis function kernel.
- Parzen: Parzen window classifier using the Euclidean distance [17]. For every training dataset, the classifier computes the width of the Parzen window by averaging the distances between objects sampled every 60 s (this procedure saved approximately seven days when computing the distances per test subject using an Intel Core i7-4600M CPU at 2.90 GHz).
- k-means1: A version of the Parzen window classifier based on k-means [2]. k-means1 classifies new objects based only on the closest center of the cluster.
- k-means2: A version of the Parzen window classifier based on k-means [18]. k-means2 classifies new objects using all of the centers of the clusters.

**Figure 1:**
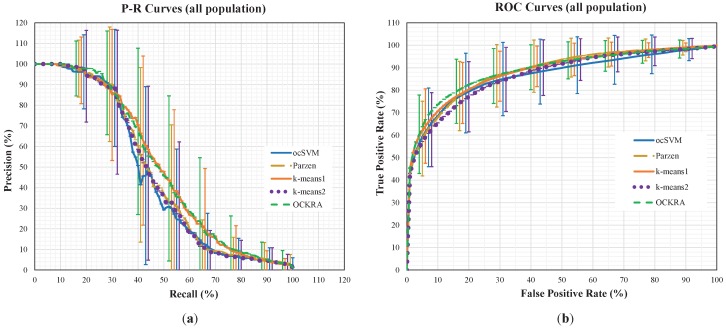
Precision-recall curves (**a**) and ROC curves (**b**) based on the average performance and standard deviation for all users.

Figure 1 shows the average P-R and ROC curves for all of the population, where the standard deviations are shown as vertical lines for each algorithm at different intervals. For the interested reader, the individual P-R and ROC curves can be accessed as Supplementary Material. The P-R curves in Figure 1 show that OCKRA and k-means1 outperformed Parzen and k-means2, and there was no significant difference between OCKRA and k-means1. The ROC curves in Figure 1 confirm the results of the P-R curves, but it can be noticed that OCKRA obtained better FPR rates between 5% and 30%.

In order to quantify the differences among the algorithms, the average of the AUC results was computed for all of the test subjects. Table 5 shows that OCKRA outperformed the other algorithms for at least 0.53% of the AUC on average. This number is small, but it has a significant impact because it means that a user will have a higher probability of assistance in a risk-prone situation. Parzen achieved the second best result in terms of AUC, but it is less suitable for running on a smartphone because it is two orders of magnitude more expensive than OCKRA (i.e., Parzen requires the full dataset to classify a new object, whereas OCKRA requires only 1000 centers of the clusters). In summary, our classifier achieved an AUC above 90% for approximately 57% of the users, which is an encouraging result.

**Figure 2:**
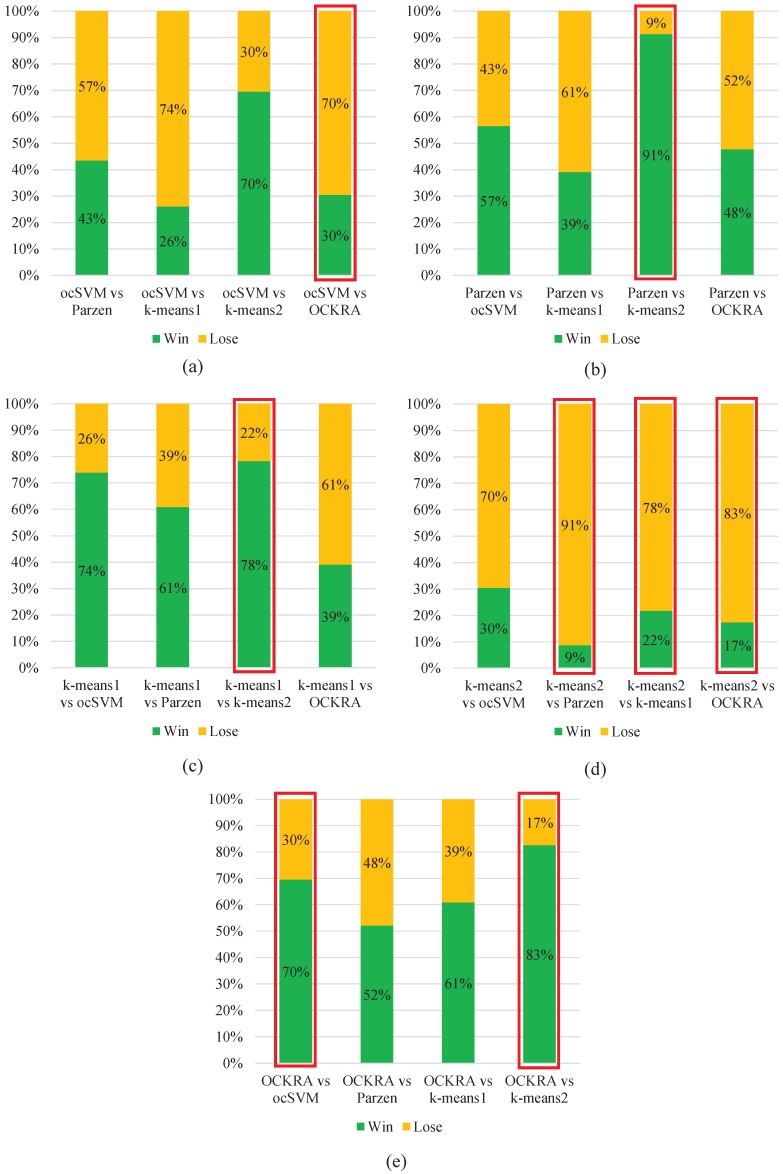
Pairwise comparisons of the algorithms based on the AUC results. AUC winning count: (**a**) ocSVM versus all; (**b**) Parzen versus all; (**c**) k-means 1 versus all; (**d**) k-means 2 versus all; (**e**) OCKRA versus all. The columns with red outer rectangles indicate significant differences according to Wilcoxon’s signed-rank test at a significance level of 0.05.

Figure 2 shows pairwise comparisons of the algorithms, which demonstrates that OCKRA outperformed the other algorithms for most of the test subjects. Furthermore, OCKRA was the only algorithm to obtain significantly higher accuracy than ocSVM according to Wilcoxon’s signed-rank test at a significance level of 0.05.

The methodology used in this study to train and test the classifier is based on subject-dependent tests; thus, the results just presented represent the potential performance of OCKRA in a production stage. By running the Kruskal–Wallis test, it has been proven that all users’ datasets are statistically different, i.e., they are not drawn from the same population; hence, using a subject-independent approach is not feasible.

## 5. Conclusions and Further Work

This study introduces a new algorithm, called OCKRA, that stands for One-Class K-means with Randomly-projected features Algorithm. OCKRA is an ensemble of one-class classifiers built over multiple projections of the dataset according to random subsets of features; OCKRA comprises 100 classifiers, each of which is built upon 10 centers computed by k-means++ with Euclidean distance.

Combining one-class classifiers has been demonstrated to outperform the classification performance obtained by single classifiers; additionally, the ensemble can be enhanced by training one or several of its classifiers with different features of the dataset [2,3,4,5,6,7,8]. Several studies have reported a wide range of applications where these techniques have been applied satisfactorily; however, none is oriented toward our area under study: personal risk detection.

Barrera-Animas et al. [1] argued that it is possible to use PRIDE, a dataset with information drawn from a number of test subjects wearing a sensor network device, to develop a personal risk detection mechanism. They showed that abnormal behavior can be automatically detected by a one-class classifier. In addition, they showed that one-class support vector machine (ocSVM) achieved the best performance and best average ranking compared with other classifiers. The present study aims to improve on the best results reported therein; i.e., to improve the anomaly detection accuracy in the context of PRIDE.

The results obtained in this study supersede those reported in [1]; therefore, OCKRA stands so far as the state-of-the-art in the context of personal risk detection. Additionally, experimental results show that OCKRA is the only algorithm that achieved significant statistical improvement over ocSVM, as shown in Figure 2.

In [1], ocSVM was compared with the following classifiers: the Parzen window classifier (PWC) using the Mahalanobis distance and two versions of PWC based on k-means with the Mahalanobis distance. The present study compared OCKRA with ocSVM [16], PWC, but this time using Euclidean distance [17], and two versions of PWC based on k-means also based on Euclidean distance [2,18]. It should be noted that all versions of PWC used in this study outperform their counterpart in [1]. In summary, the authors have knowledge of eight different classifiers tested on the same one-class dataset.

It remains an open question, beyond the scope of this paper, to compare OCKRA with other one-class algorithms in a different context; that is, using a different dataset. Furthermore, as discussed in [1], it is difficult to find open-access datasets posing a one-class detection problem.

A publicly-available one-class dataset that could be a good candidate for comparison purposes is WUIL (the Windows-Users and -Intruder simulations Logs) [27]. However, preliminary tests on WUIL have led to the conclusion that classifiers based on the Mahalanobis distance perform better on WUIL than those based on Euclidean distance, whereas in the context of PRIDE, our results show exactly the opposite. Due to the scarcity of datasets posing a one-class classification problem, a common practice in the research community is to transform a “multi-class” dataset into a “one-class” dataset. However, the authors have so far refrained from adopting this procedure since modifying the problem domain amounts to creating a new domain that often departs from the original nature of the problem.

The main contribution of this study is the introduction of OCKRA, a new algorithm which is an ensemble specially designed for one-class classification, which is highly suitable for personal risk detection based on wearable sensors.

Results showed that OCKRA outperformed the other algorithms for at least 0.53% of the AUC on average. This number is small, but it has a significant impact because it means that a user will have a higher probability of being assisted in time if they encounter a risk-prone situation. The classifier achieved an AUC above 90% for more than 57% of the users, which is an encouraging result because it supports the hypothesis that abnormal behavior can be detected automatically.

A promising approach to consider in future work is to combine the proposed ensemble with other classifiers that take into account temporal relationships among consecutive events. In addition, another approach will be explored that updates the ensemble knowledge continuously whenever it misclassifies normal behavior as abnormal (i.e., when it generates a false alarm).

Furthermore, due to the high amount of raw data collected by users, the possibility of implementing mechanisms based on information fusion [28] will be considered, with the aims of both decreasing the amount of data stored/transmitted and improving the classification process.

## Figures and Tables

**Table 1:** Summary and comparison of the related research.

Reference	Feature	Object	Ensemble Type	Pruning	Application Domain
	Selection	Selection		Technique	
Tax and Duin [2]	*√*	×	C${}_{1}$ and C${}_{2}$	×	Handwritten recognition
Juszczak and Duin [3]	*√*	×	C${}_{1}$	×	Missing feature values
Nanni [4]	*√*	×	C${}_{1}$ and C${}_{2}$	×	Online signature verification system
Biggio et al. [5]	*√*	×	C${}_{1}$	×	Adversarial classification task
Cheplygina and Tax [6]	*√*	×	C${}_{1}$	*√*	Improvement of one-class classifiers
Krawczyk [8]	*√*	×	C${}_{1}$	*√*	Improvement of one-class classifiers
Medina-Pérez et al. [13]	×	*√*	C${}_{1}$	×	Masquerader detection
OCKRA	*√*	*√*	C${}_{1}$	×	Personal risk detection

Note: C${}_{1}$ denotes an ensemble built from multiple instances of the same base classifier; C${}_{2}$ denotes an ensemble built from multiple instances of different base classifiers or different single classifiers.

**Table 2:** Sensor descriptions.

Sensor	Description	Frequency
Accelerometer	Provides X, Y and Z acceleration in g units. 1 g = 9.81 m per second squared (m/s${}^{2}$).	8 Hz
Gyroscope	Provides X, Y and Z angular velocity in degrees per second (${}^{\circ }$/s) units.	8 Hz
Distance	Provides the total distance in centimeters, current speed in centimeters per second (cm/s), current pace in milliseconds per meter (ms/m).	1 Hz
Heart Rate	Provides the number of beats per minute, also indicates if the heart rate sensor is fully locked onto the wearer’s heart rate	1 Hz
Pedometer	Provides the total number of steps the user has taken.	1 Hz
Skin Temperature	Provides the current skin temperature of the user in degrees Celsius.	33 MHz
UV	Provides the current ultraviolet radiation exposure intensity (none, low, medium, high, very high)	16 MHz
Calories	Provides the total number of calories burned by the user.	1 Hz

**Table 3:** Feature vector structure (Fields 1 to 18).

Gyroscope Accelerometer						Gyroscope Angular Velocity						Accelerometer					
X Axis		Y Axis		Z Axis		X Axis		Y Axis		Z Axis		X Axis		Y Axis		Z Axis	
$\bar{x}$	s	$\bar{x}$	s	$\bar{x}$	s	$\bar{x}$	s	$\bar{x}$	s	$\bar{x}$	s	$\bar{x}$	s	$\bar{x}$	s	$\bar{x}$	s
1	2	3	4	5	6	7	8	9	10	11	12	13	14	15	16	17	18

**Table 4:** Feature vector structure (Fields 19 to 26).

Heart Rate	Skin Temperature	Pace	Speed	UV	Δ Pedometer	Δ Distance	Δ Calories
19	20	21	22	23	24	25	26

**Table 5:** Area (percentage) under the curve for TPR versus FPR.

Test Subject	ocSVM	Parzen	k-Means1	k-Means2	OCKRA
TS 1	97.3	96.6	98.5	96.6	98.8
TS 2	94.5	95.4	95.5	92.5	95.7
TS 3	87.4	88.3	90.1	87.1	91.2
TS 4	83.9	83.6	89.9	81.9	88.2
TS 5	80.8	92.3	84.3	91.2	90.2
TS 6	96.1	95.6	97.0	96.0	98.2
TS 7	69.4	77.0	78.0	76.8	79.2
TS 8	93.8	93.5	90.0	91.4	92.4
TS 9	95.3	93.2	91.0	89.8	92.7
TS 10	94.0	93.7	86.9	93.3	93.7
TS 11	93.4	92.7	89.5	91.3	90.9
TS 12	74.6	76.5	80.1	76.0	80.3
TS 13	75.8	79.9	80.1	76.7	80.5
TS 14	78.0	83.8	82.4	82.2	81.9
TS 15	93.8	93.0	94.1	90.6	94.5
TS 16	83.2	88.3	88.1	87.1	87.9
TS 17	98.1	98.2	95.7	97.8	98.0
TS 18	89.1	89.3	89.5	87.0	86.9
TS 19	89.4	88.2	91.4	88.0	89.6
TS 20	90.5	91.5	92.7	87.3	92.2
TS 21	98.4	95.7	98.0	94.9	97.9
TS 22	78.3	79.6	79.3	76.7	79.2
TS 23	53.0	71.0	73.8	64.4	68.9
**Average**	**86.44**	**88.56**	**88.52**	**86.81**	**89.09**

## Supplementary Materials

The following are available online at http://www.mdpi.com/1424-8220/16/10/1619/s1.

## Abbreviations

The following abbreviations are used in this manuscript:

ACDS	Anomaly conditions dataset
AUC	Area under the curve
CPU	Central processing unit
CSV	Comma-separated values
FPR	False positive detection rate
FTP	File transfer protocol
GPS	Global positioning system
LibSVM	Support vector machine library
MCYT	Ministerio de Ciencia y Tecnología (Spanish Ministry of Science and Technology)
NCDS	Normal conditions dataset
OCKRA	One-Class K-means with Randomly-projected features Algorithm
ocSVM	One-class support vector machine
PRIDE	Personal RIsk DEtection
P-R	Precision-recall
RAM	Random access memory
ROC	Receiver operating characteristic
RSM	Random subspace method
SDK	Software development kit
SIAM	Society for Industrial and Applied Mathematics
SVM	Support vector machine
TPR	True positive detection rate
TREC	Text REtrieval Conference
UCI	University of California Irvine

## Appendix A. PRIDE Dataset

PRIDE Dataset: $\bar{X}$ and s for All Test Subject Features are shown in Table A1, Table A2, Table A3, Table A4, Table A5 and Table A6.

**Table A1:** Normal conditions dataset. Part I.

	No. of Records	Gyroscope Accelerometer			Gyroscope Angular Velocity		
		X Axis $\bar{x}$ (s)	Y Axis $\bar{x}$ (s)	Z Axis $\bar{x}$ (s)	X Axis $\bar{x}$ (s)	Y Axis $\bar{x}$ (s)	Z Axis $\bar{x}$ (s)
TS 1	466,175	−0.066 (0.479)	0.282 (0.491)	0.312 (0.573)	0.346 (13.712)	−0.644 (9.379)	−0.894 (12.138)
TS 2	314,975	−0.060 (0.493)	−0.349 (0.531)	−0.201 (0.551)	−0.646 (20.150)	0.837 (15.469)	−0.090 (16.640)
TS 3	341,645	−0.079 (0.439)	−0.296 (0.452)	0.493 (0.508)	−0.298 (18.403)	−0.120 (12.021)	0.053 (15.084)
TS 4	373,019	0.051 (0.567)	−0.347 (0.424)	0.359 (0.483)	−0.702 (16.034)	−0.411 (11.139)	0.174 (12.828)
TS 5	296,270	−0.094 (0.487)	0.321 (0.473)	0.381 (0.520)	−0.711 (18.799)	0.463 (12.088)	−0.398 (14.399)
TS 6	328,976	−0.214 (0.432)	0.359 (0.435)	0.463 (0.488)	−0.606 (14.530)	−0.431 (9.046)	−0.062 (11.104)
TS 7	288,386	0.024 (0.519)	−0.398 (0.448)	0.318 (0.512)	−0.083 (19.638)	−0.254 (13.010)	−0.945 (16.512)
TS 8	397,320	−0.128 (0.543)	0.263 (0.492)	0.387 (0.471)	−0.412 (14.073)	−0.157 (8.302)	−0.023 (9.557)
TS 9	336,474	−0.190 (0.511)	−0.267 (0.563)	−0.149 (0.541)	0.377 (12.486)	−0.671 (10.083)	−0.879 (10.745)
TS 10	251,383	−0.123 (0.493)	0.334 (0.424)	0.566 (0.360)	−0.344 (13.887)	−0.079 (9.187)	−0.036 (13.403)
TS 11	442,304	−0.032 (0.412)	−0.299 (0.491)	−0.262 (0.640)	−0.601 (15.472)	−0.287 (10.329)	0.058 (11.972)
TS 12	243,701	0.016 (0.485)	0.265 (0.432)	0.520 (0.474)	−0.735 (19.527)	0.087 (12.183)	−0.373 (14.832)
TS 13	431,496	0.019 (0.451)	−0.321 (0.534)	0.225 (0.598)	−0.515 (15.429)	−0.253 (10.246)	0.018 (11.710)
TS 14	160,975	−0.082 (0.498)	0.430 (0.373)	−0.276 (0.582)	0.262 (24.231)	−0.861 (15.893)	−1.255 (19.733)
TS 15	302,863	−0.178 (0.448)	0.287 (0.540)	0.220 (0.588)	−0.722 (19.533)	−0.382 (14.578)	0.030 (15.678)
TS 16	327,804	0.001 (0.481)	0.379 (0.430)	−0.503 (0.428)	−0.832 (18.842)	−0.017 (13.758)	−0.002 (16.473)
TS 17	133,795	−0.094 (0.396)	0.168 (0.628)	−0.356 (0.536)	0.259 (11.996)	−0.627 (7.500)	−0.948 (9.317)
TS 18	335,424	−0.021 (0.511)	−0.376 (0.492)	−0.225 (0.546)	−0.725 (12.759)	0.548 (8.500)	−0.170 (11.065)
TS 19	400,906	0.001 (0.515)	0.229 (0.412)	0.537 (0.480)	−0.844 (19.031)	−0.721 (12.126)	−0.056 (15.083)
TS 20	300,461	−0.048 (0.458)	0.122 (0.550)	0.442 (0.526)	−1.281 (16.373)	−0.889 (10.710)	0.106 (13.007)
TS 21	359,603	−0.048 (0.421)	0.118 (0.567)	0.242 (0.647)	−0.704 (11.529)	0.534 (8.535)	−0.289 (10.951)
TS 22	373,783	−0.033 (0.549)	0.257 (0.535)	0.151 (0.576)	−0.028 (18.364)	−0.347 (14.401)	−0.903 (15.219)
TS 23	209,128	−0.082 (0.519)	0.060 (0.665)	0.039 (0.545)	−0.617 (18.176)	−0.020 (12.978)	0.084 (14.793)

**Table A2:** Normal conditions dataset. Part II.

	Accelerometer			Heart Rate	Skin Temperature	Pace
	X Axis $\bar{x}$ (s)	Y Axis $\bar{x}$ (s)	Z Axis $\bar{x}$ (s)	$\bar{x}$ (s)	$\bar{x}$ (s)	$\bar{x}$ (s)
TS 1	−0.066 (0.479)	0.282 (0.491)	0.312 (0.573)	71.856 (10.254)	33.553 (1.300)	47.613 (249.390)
TS 2	−0.059 (0.493)	−0.349 (0.531)	−0.201 (0.551)	64.426 (10.274)	32.821 (1.412)	134.718 (431.197)
TS 3	−0.079 (0.439)	−0.296 (0.452)	0.493 (0.508)	71.488 (8.425)	31.885 (1.789)	74.004 (319.042)
TS 4	0.051 (0.567)	−0.347 (0.424)	0.359 (0.483)	68.335 (10.309)	32.127 (2.239)	49.351 (271.310)
TS 5	−0.094 (0.487)	0.321 (0.473)	0.381 (0.520)	68.911 (9.593)	33.381 (2.001)	65.316 (301.010)
TS 6	−0.214 (0.432)	0.359 (0.435)	0.463 (0.488)	66.160 (11.398)	32.749 (1.144)	49.332 (248.829)
TS 7	0.024 (0.519)	−0.398 (0.447)	0.318 (0.512)	74.341 (9.454)	33.184 (3.004)	156.612 (438.505)
TS 8	−0.128 (0.543)	0.263 (0.491)	0.387 (0.471)	65.663 (11.600)	31.970 (1.880)	43.232 (219.687)
TS 9	−0.190 (0.511)	−0.267 (0.563)	−0.149 (0.541)	81.414 (11.777)	34.166 (1.571)	46.143 (247.978)
TS 10	−0.123 (0.493)	0.334 (0.424)	0.566 (0.360)	77.573 (14.253)	32.970 (1.239)	40.556 (220.579)
TS 11	−0.032 (0.412)	−0.299 (0.492)	−0.262 (0.640)	72.384 (10.791)	34.237 (1.426)	40.066 (232.637)
TS 12	0.016 (0.485)	0.265 (0.432)	0.520 (0.474)	71.344 (9.123)	38.917 (1.942)	65.041 (296.546)
TS 13	0.019 (0.451)	−0.321 (0.534)	0.225 (0.598)	71.832 (15.471)	31.981 (2.379)	72.149 (296.384)
TS 14	−0.082 (0.498)	0.430 (0.373)	−0.276 (0.582)	70.741 (7.681)	32.512 (1.615)	102.405 (375.538)
TS 15	−0.178 (0.448)	0.287 (0.540)	0.220 (0.588)	71.081 (8.969)	32.926 (2.354)	80.668 (340.809)
TS 16	0.001 (0.481)	0.379 (0.430)	−0.503 (0.428)	73.199 (10.221)	33.224 (2.119)	84.804 (339.817)
TS 17	−0.094 (0.396)	0.168 (0.628)	−0.356 (0.536)	61.294 (11.812)	34.141 (1.331)	70.054 (325.963)
TS 18	−0.021 (0.511)	−0.376 (0.492)	−0.225 (0.546)	73.254 (10.305)	41.339 (1.881)	69.961 (284.993)
TS 19	0.001 (0.515)	0.229 (0.412)	0.537 (0.481)	67.203 (9.325)	31.956 (2.218)	95.774 (365.087)
TS 20	−0.048 (0.458)	0.122 (0.550)	0.442 (0.526)	77.566 (16.511)	31.969 (2.306)	59.234 (287.112)
TS 21	−0.048 (0.421)	0.118 (0.567)	0.242 (0.647)	73.858 (6.150)	37.665 (2.952)	31.313 (218.225)
TS 22	−0.033 (0.549)	0.257 (0.535)	0.151 (0.576)	72.621 (7.315)	31.056 (3.128)	71.868 (311.424)
TS 23	−0.082 (0.519)	0.060 (0.665)	0.039 (0.544)	74.748 (19.082)	32.344 (4.198)	57.822 (262.482)

**Table A3:** Normal conditions dataset. Part III.

	Speed $\bar{x}$ (s)	UV $\bar{x}$ (s)	Δ Pedometer $\bar{x}$ (s)	Δ Distance $\bar{x}$ (s)	Δ Calories $\bar{x}$ (s)
TS 1	4.992 (23.964)	0.022 (0.148)	0.068 (0.513)	5.277 (40.239)	0.026 (0.810)
TS 2	11.247 (33.578)	0.020 (0.183)	0.180 (5.749)	14.001 (449.963)	0.037 (1.902)
TS 3	7.146 (28.243)	0.036 (0.203)	0.107 (2.468)	8.187 (183.953)	0.034 (1.975)
TS 4	3.871 (19.806)	0.035 (0.184)	0.087 (8.173)	6.946 (653.910)	0.030 (1.789)
TS 5	5.678 (24.098)	0.038 (0.203)	0.111 (6.601)	8.859 (527.082)	0.037 (1.954)
TS 6	5.123 (23.836)	0.020 (0.138)	0.087 (3.533)	6.788 (308.571)	0.037 (2.059)
TS 7	17.295 (46.119)	0.034 (0.206)	0.298 (26.685)	24.777 (2186.446)	0.042 (1.936)
TS 8	5.117 (24.167)	0.040 (0.195)	0.088 (4.579)	6.993 (362.702)	0.034 (1.597)
TS 9	4.311 (21.449)	0.027 (0.173)	0.068 (1.205)	4.804 (85.353)	0.022 (0.797)
TS 10	5.343 (26.415)	0.025 (0.156)	0.101 (6.972)	7.950 (550.756)	0.046 (3.286)
TS 11	3.477 (18.744)	0.000 (0.000)	0.057 (0.659)	3.841 (43.986)	0.024 (0.807)
TS 12	5.966 (25.317)	0.046 (0.226)	0.105 (5.378)	8.240 (417.039)	0.040 (2.834)
TS 13	9.248 (35.681)	0.056 (0.268)	0.253 (80.693)	11.237 (463.311)	0.042 (1.507)
TS 14	8.549 (29.013)	0.039 (0.193)	0.320 (31.097)	19.210 (1361.480)	0.055 (4.229)
TS 15	6.909 (27.264)	0.022 (0.183)	0.198 (12.125)	15.908 (970.020)	0.044 (1.611)
TS 16	7.687 (28.507)	0.011 (0.105)	0.929 (469.171)	8.790 (208.055)	0.035 (1.633)
TS 17	5.485 (23.726)	0.004 (0.060)	0.282 (31.805)	22.152 (2507.279)	0.062 (5.433)
TS 18	8.582 (31.890)	0.001 (0.029)	0.120 (2.264)	9.411 (177.234)	0.035 (2.723)
TS 19	8.361 (29.700)	0.040 (0.205)	0.261 (74.531)	12.155 (856.181)	0.033 (1.216)
TS 20	5.767 (25.677)	0.027 (0.172)	0.099 (5.606)	7.917 (447.581)	0.045 (2.562)
TS 21	2.517 (16.320)	0.000 (0.000)	0.914 (515.916)	3.794 (201.550)	0.410 (231.157)
TS 22	7.137 (28.469)	0.043 (0.227)	0.126 (7.354)	9.356 (557.696)	0.025 (1.090)
TS 23	11.618 (47.721)	0.022 (0.148)	0.274 (17.692)	21.581 (1330.140)	0.045 (2.482)

**Table A4:** Anomaly conditions dataset. Part I.

	No. of Records	Gyroscope Accelerometer			Gyroscope Angular Velocity		
		X Axis $\bar{x}$ (s)	Y Axis $\bar{x}$ (s)	Z Axis $\bar{x}$ (s)	X Axis $\bar{x}$ (s)	Y Axis $\bar{x}$ (s)	Z Axis $\bar{x}$ (s)
TS 1	770	0.113 (0.619)	−0.679 (0.414)	0.017 (0.417)	−0.846 (40.900)	−1.102 (38.233)	0.524 (33.024)
TS 2	608	0.384 (0.501)	−0.699 (0.492)	0.114 (0.642)	2.209 (50.987)	2.188 (31.660)	−3.077 (35.142)
TS 3	494	0.339 (0.803)	−0.655 (0.673)	0.374 (0.410)	−12.002 (59.791)	−4.086 (32.065)	2.214 (33.438)
TS 4	708	0.431 (0.609)	−0.346 (0.387)	0.324 (0.489)	−3.294 (42.243)	−2.153 (34.449)	0.413 (33.770)
TS 5	792	0.586 (0.783)	0.532 (0.525)	0.161 (0.349)	2.098 (29.158)	−2.664 (27.008)	−0.408 (38.388)
TS 6	674	0.457 (0.669)	0.571 (0.658)	0.560 (0.446)	1.093 (47.790)	−1.054 (32.493)	0.516 (40.079)
TS 7	770	0.411 (0.685)	−0.635 (0.606)	0.298 (0.389)	1.095 (41.332)	1.919 (35.214)	−2.004 (42.678)
TS 8	724	0.580 (0.838)	0.832 (0.657)	−0.214 (0.617)	2.072 (39.552)	−10.207 (39.374)	−1.571 (36.128)
TS 9	566	0.086 (0.647)	−0.399 (0.556)	0.449 (0.350)	2.317 (40.801)	−0.828 (36.996)	−5.515 (42.064)
TS 10	767	0.299 (0.517)	0.321 (0.818)	−0.023 (0.532)	2.590 (37.810)	1.085 (25.954)	−0.927 (30.640)
TS 11	647	0.365 (0.739)	−0.236 (0.626)	0.231 (0.575)	−2.795 (45.353)	5.866 (32.214)	3.660 (49.090)
TS 12	679	0.740 (0.716)	0.058 (0.600)	0.454 (0.466)	−0.114 (41.168)	−1.030 (35.996)	−2.367 (36.503)
TS 13	485	0.491 (0.826)	−0.549 (0.526)	0.030 (0.494)	−0.647 (41.101)	−4.246 (41.316)	2.940 (41.405)
TS 14	1,066	0.464 (0.656)	0.491 (0.502)	0.108 (0.507)	−5.939 (40.240)	5.508 (32.998)	−1.785 (33.435)
TS 15	881	0.245 (0.593)	0.799 (0.687)	−0.095 (0.430)	7.482 (48.483)	2.926 (37.574)	−0.901 (43.749)
TS 16	578	0.560 (0.796)	0.809 (0.667)	−0.130 (0.475)	−7.245 (39.853)	7.422 (31.144)	0.075 (37.856)
TS 17	941	0.194 (0.525)	0.689 (0.534)	0.295 (0.501)	1.151 (45.200)	0.685 (25.359)	−0.138 (29.349)
TS 18	904	0.320 (0.574)	−0.656 (0.339)	0.213 (0.428)	−0.638 (38.582)	2.106 (27.732)	−3.557 (33.302)
TS 19	769	0.542 (0.800)	0.785 (0.506)	−0.082 (0.371)	0.284 (35.969)	−3.791 (31.353)	−3.904 (36.100)
TS 20	586	0.655 (1.105)	0.820 (0.855)	0.290 (0.415)	3.980 (45.278)	−7.457 (33.883)	−1.901 (43.004)
TS 21	423	0.127 (0.480)	0.854 (0.591)	0.061 (0.529)	5.873 (42.590)	3.025 (36.399)	−2.729 (39.754)
TS 22	584	0.231 (0.674)	0.684 (0.657)	−0.095 (0.726)	9.598 (46.259)	−2.294 (59.213)	3.746 (57.355)
TS 23	854	0.389 (0.598)	0.841 (0.485)	−0.199 (0.468)	9.798 (51.226)	−2.865 (31.501)	1.634 (40.658)

**Table A5:** Anomaly conditions dataset. Part II.

	Accelerometer			Heart Rate	Skin Temperature	Pace
	X Axis $\bar{x}$ (s)	Y Axis $\bar{x}$ (s)	Z Axis	$\bar{x}$ (s)	$\bar{x}$ (s)	$\bar{x}$ (s)
TS 1	0.110 (0.622)	−0.680 (0.411)	0.015 (0.417)	97.097 (22.148)	31.739 (0.429)	258.291 (371.325)
TS 2	0.395 (0.482)	−0.696 (0.511)	0.156 (0.670)	80.655 (6.793)	29.113 (1.196)	460.569 (419.805)
TS 3	0.338 (0.805)	−0.655 (0.667)	0.374 (0.411)	87.263 (17.918)	28.942 (1.791)	287.767 (418.495)
TS 4	0.431 (0.611)	−0.346 (0.388)	0.324 (0.491)	82.602 (15.916)	28.941 (1.645)	558.905 (633.728)
TS 5	0.603 (0.781)	0.538 (0.528)	0.154 (0.352)	81.412 (11.368)	33.933 (0.829)	339.201 (542.872)
TS 6	0.457 (0.668)	0.569 (0.652)	0.555 (0.450)	85.220 (14.215)	29.616 (0.402)	246.426 (371.220)
TS 7	0.412 (0.685)	−0.635 (0.605)	0.297 (0.390)	76.544 (6.768)	29.921 (1.307)	393.745 (574.313)
TS 8	0.579 (0.837)	0.818 (0.653)	−0.215 (0.615)	79.148 (8.492)	28.358 (0.921)	221.006 (310.291)
TS 9	0.085 (0.643)	−0.399 (0.555)	0.448 (0.347)	79.936 (12.531)	30.151 (1.243)	160.900 (259.476)
TS 10	0.298 (0.518)	0.321 (0.817)	−0.023 (0.532)	81.675 (11.959)	30.589 (1.417)	221.785 (413.279)
TS 11	0.365 (0.739)	−0.236 (0.625)	0.232 (0.573)	86.611 (20.806)	31.249 (1.198)	206.471 (305.513)
TS 12	0.746 (0.740)	0.054 (0.607)	0.446 (0.482)	79.031 (8.765)	39.856 (1.977)	262.915 (472.168)
TS 13	0.492 (0.829)	−0.550 (0.528)	0.030 (0.493)	79.563 (8.671)	31.597 (0.993)	230.324 (435.552)
TS 14	0.466 (0.661)	0.490 (0.494)	0.102 (0.501)	83.398 (14.065)	31.157 (0.984)	251.290 (415.069)
TS 15	0.253 (0.597)	0.799 (0.673)	−0.085 (0.433)	79.748 (11.037)	27.039 (0.673)	330.247 (538.912)
TS 16	0.564 (0.799)	0.811 (0.669)	−0.131 (0.475)	88.540 (20.952)	33.407 (0.918)	216.730 (353.419)
TS 17	0.189 (0.516)	0.690 (0.533)	0.310 (0.499)	81.963 (16.540)	27.289 (0.985)	120.472 (243.084)
TS 18	0.320 (0.573)	−0.655 (0.342)	0.213 (0.428)	93.290 (24.697)	37.815 (2.559)	183.941 (356.975)
TS 19	0.542 (0.801)	0.787 (0.502)	−0.083 (0.373)	70.281 (8.279)	24.584 (2.809)	242.893 (432.227)
TS 20	0.654 (1.105)	0.825 (0.864)	0.289 (0.413)	101.604 (31.635)	29.182 (0.738)	164.817 (294.219)
TS 21	0.127 (0.479)	0.854 (0.594)	0.061 (0.528)	77.934 (11.546)	30.818 (1.186)	240.584 (259.975)
TS 22	0.253 (0.670)	0.688 (0.655)	−0.090 (0.743)	77.926 (7.718)	27.729 (0.881)	278.907 (447.636)
TS 23	0.398 (0.600)	0.838 (0.487)	−0.186 (0.446)	75.689 (6.032)	28.225 (1.819)	221.013 (432.126)

**Table A6:** Anomaly conditions dataset. Part III.

	Speed $\bar{x}$ (s)	UV $\bar{x}$ (s)	Δ Pedometer $\bar{x}$ (s)	Δ Distance$\bar{x}$ (s)	Δ Calories $\bar{x}$ (s)
TS 1	118.190 (133.248)	0.118 (0.323)	1.164 (1.662)	123.790 (178.250)	0.078 (0.268)
TS 2	176.948 (122.629)	0.523 (0.500)	1.714 (1.680)	176.194 (181.270)	0.094 (0.292)
TS 3	134.763 (160.947)	0.223 (0.516)	1.279 (1.846)	142.591 (229.427)	0.071 (0.257)
TS 4	77.801 (92.240)	0.097 (0.297)	0.893 (1.601)	82.054 (159.729)	0.055 (0.228)
TS 5	104.194 (138.165)	0.665 (0.472)	0.990 (1.782)	105.869 (190.114)	0.037 (0.188)
TS 6	144.616 (157.949)	0.405 (0.491)	1.315 (1.788)	151.721 (210.877)	0.042 (0.200)
TS 7	116.610 (145.402)	0.553 (0.832)	1.047 (1.710)	121.081 (203.444)	0.032 (0.177)
TS 8	140.436 (158.209)	0.394 (0.489)	1.199 (1.776)	141.738 (221.937)	0.033 (0.179)
TS 9	117.992 (149.526)	0.498 (0.500)	1.155 (1.712)	127.212 (193.314)	0.023 (0.150)
TS 10	85.001 (126.758)	0.248 (0.432)	0.866 (1.616)	95.557 (180.922)	0.031 (0.174)
TS 11	85.121 (115.315)	0.233 (0.423)	0.989 (1.573)	94.414 (152.147)	0.039 (0.193)
TS 12	124.980 (162.112)	0.236 (0.492)	1.037 (1.939)	125.973 (235.163)	0.037 (0.188)
TS 13	121.590 (164.312)	0.243 (0.598)	1.010 (1.775)	128.878 (238.527)	0.035 (0.184)
TS 14	88.249 (126.044)	0.067 (0.249)	0.797 (1.455)	86.081 (170.038)	0.034 (0.181)
TS 15	109.222 (152.789)	0.314 (0.465)	0.981 (1.711)	114.547 (205.342)	0.034 (0.181)
TS 16	139.817 (169.673)	0.351 (0.478)	1.128 (1.678)	141.412 (222.837)	0.059 (0.235)
TS 17	71.153 (125.804)	0.000 (0.000)	0.618 (1.416)	72.259 (169.026)	0.052 (0.222)
TS 18	85.978 (125.400)	0.198 (0.399)	0.772 (1.468)	86.354 (168.715)	0.063 (0.243)
TS 19	106.29 (155.192)	0.000 (0.000)	0.830 (1.634)	106.052 (216.716)	0.031 (0.174)
TS 20	132.802 (177.826)	0.152 (0.359)	1.017 (1.675)	133.014 (237.409)	0.087 (0.282)
TS 21	148.603 (142.036)	0.000 (0.000)	1.556 (1.835)	161.296 (207.782)	0.026 (0.159)
TS 22	108.405 (131.115)	0.000 (0.000)	1.058 (2.150)	112.277 (247.873)	0.024 (0.153)
TS 23	81.359 (131.804)	0.354 (0.478)	0.721 (1.517)	83.218 (187.737)	0.020 (0.140)
