# Supplementary material for: Ensemble of One-Class Classifiers for Personal Risk Detection Based on Wearable Sensor Data
Source: Sensors (Basel). 2016 Sep 29;16(10):1619. doi: 10.3390/s16101619 (PMC5087407; doi:10.3390/s16101619)
Supplement: Supplementary file 1 [file sensors-16-01619-s001.zip › SupplementaryFiles/Individual ROC Curves.pdf]

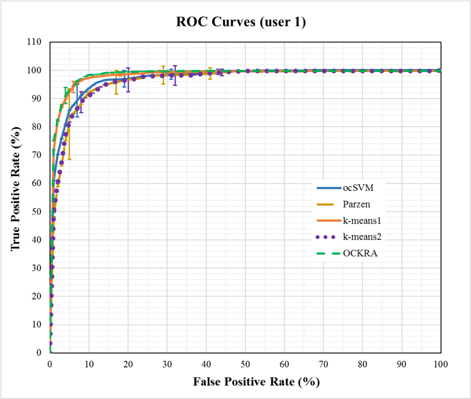
ROC Curves (user 1)
True Positive Rate (%)
False Positive Rate (%)
ocSVM
Parzen
k-means1
k-means2
OCKRA

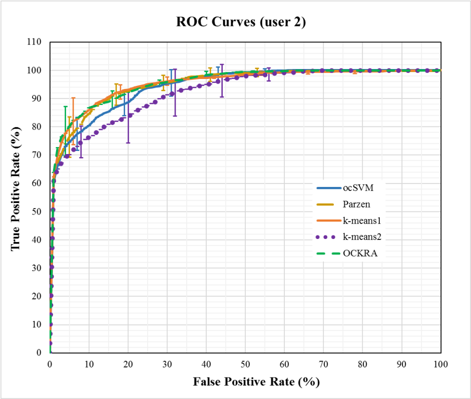
ROC Curves (user 2)
True Positive Rate (%)
False Positive Rate (%)
ocSVM
Parzen
k-means1
k-means2
OCKRA

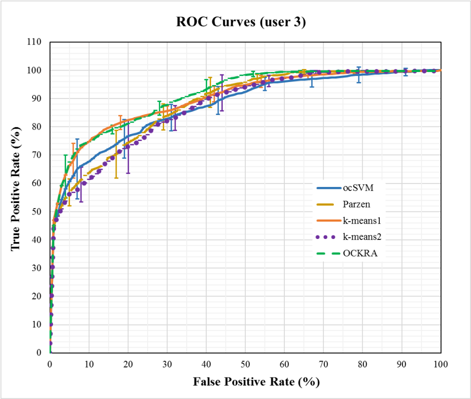
ROC Curves (user 3)
True Positive Rate (%)
False Positive Rate (%)
ocSVM
Parzen
k-means1
k-means2
OCKRA

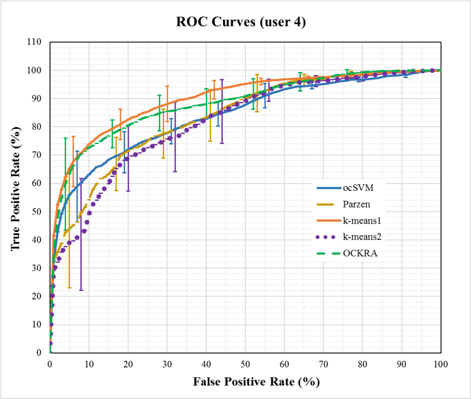
ROC Curves (user 4)
True Positive Rate (%)
False Positive Rate (%)
ocSVM
Parzen
k-means1
k-means2
OCKRA

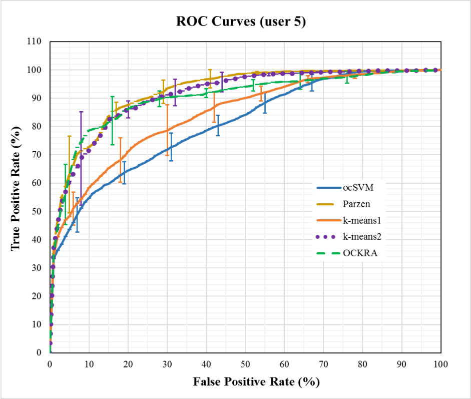
ROC Curves (user 5)
True Positive Rate (%)
False Positive Rate (%)
ocSVM
Parzen
k-means1
k-means2
OCKRA

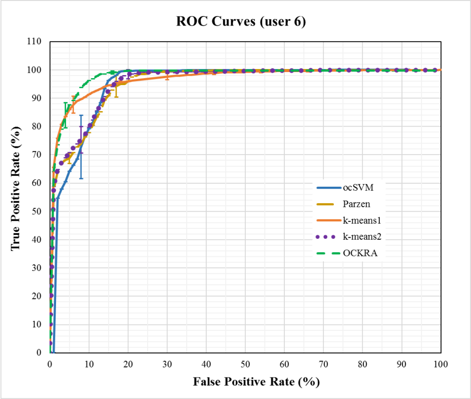
ROC Curves (user 6)
True Positive Rate (%)
False Positive Rate (%)
ocSVM
Parzen
k-means1
k-means2
OCKRA

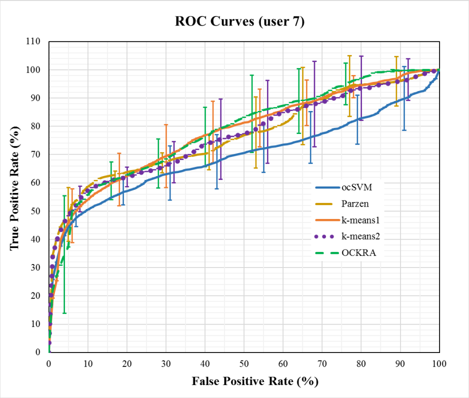
ROC Curves (user 7)
True Positive Rate (%)
False Positive Rate (%)
0 10 20 30 40 50 60 70 80 90 100 110
0 10 20 30 40 50 60 70 80 90 100
ocSVM
Parzen
k-means1
k-means2
OCKRA

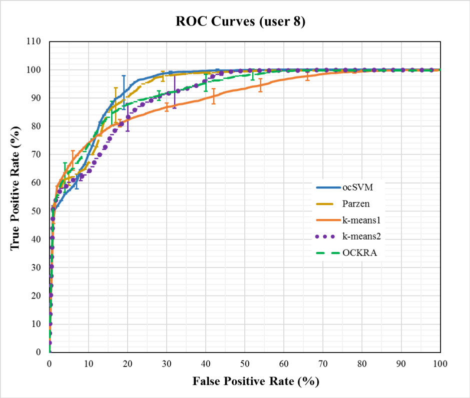
ROC Curves (user 8)
True Positive Rate (%)
False Positive Rate (%)
0 10 20 30 40 50 60 70 80 90 100 110
0 10 20 30 40 50 60 70 80 90 100
ocSVM
Parzen
k-means1
k-means2
OCKRA

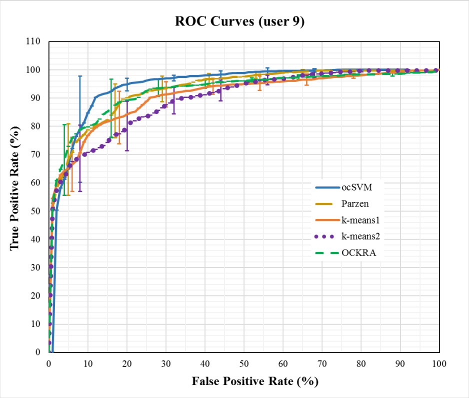
ROC Curves (user 9)
True Positive Rate (%)
False Positive Rate (%)
0 10 20 30 40 50 60 70 80 90 100 110
0 10 20 30 40 50 60 70 80 90 100
ocSVM
Parzen
k-means1
k-means2
OCKRA

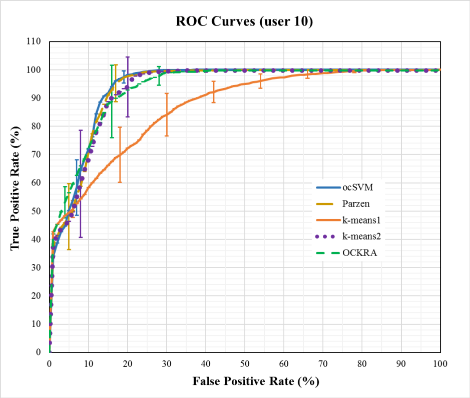
ROC Curves (user 10)
True Positive Rate (%)
False Positive Rate (%)
0 10 20 30 40 50 60 70 80 90 100 110
0 10 20 30 40 50 60 70 80 90 100
ocSVM
Parzen
k-means1
k-means2
OCKRA

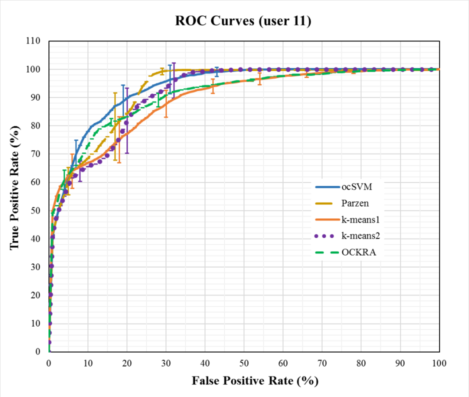
ROC Curves (user 11)
True Positive Rate (%)
False Positive Rate (%)
0 10 20 30 40 50 60 70 80 90 100 110
0 10 20 30 40 50 60 70 80 90 100
ocSVM
Parzen
k-means1
k-means2
OCKRA

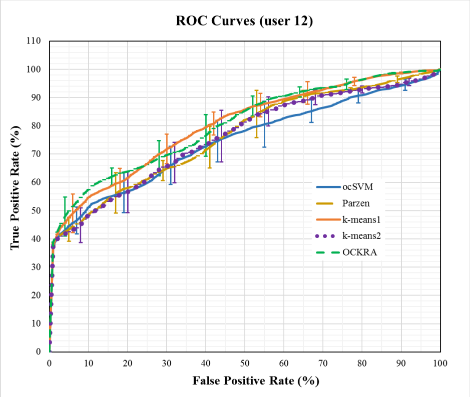
ROC Curves (user 12)
True Positive Rate (%)
False Positive Rate (%)
0 10 20 30 40 50 60 70 80 90 100 110
0 10 20 30 40 50 60 70 80 90 100
ocSVM
Parzen
k-means1
k-means2
OCKRA

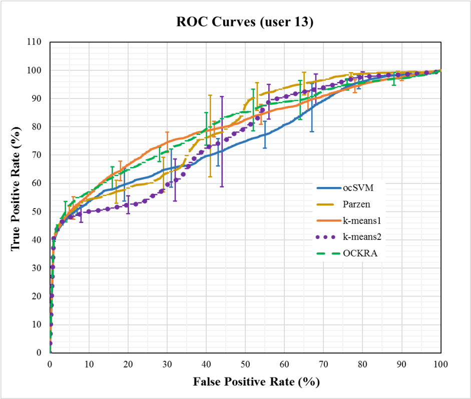
ROC Curves (user 13)
True Positive Rate (%)
False Positive Rate (%)
ocSVM
Parzen
k-means1
k-means2
OCKRA

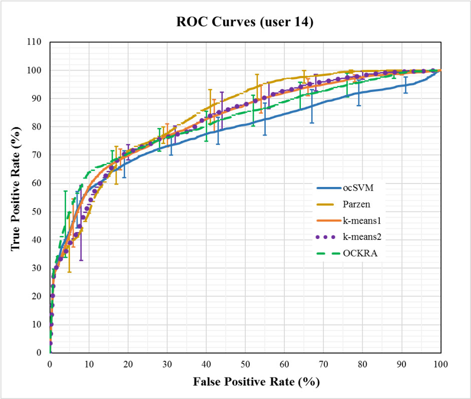
ROC Curves (user 14)
True Positive Rate (%)
False Positive Rate (%)
ocSVM
Parzen
k-means1
k-means2
OCKRA

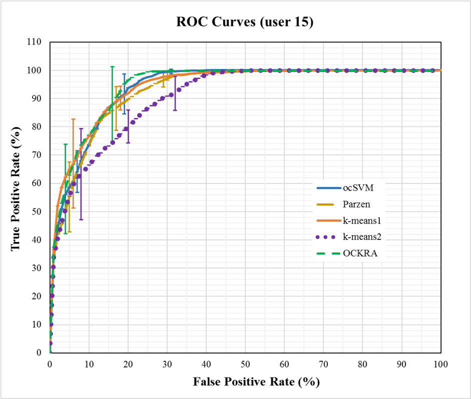
ROC Curves (user 15)
True Positive Rate (%)
False Positive Rate (%)
ocSVM
Parzen
k-means1
k-means2
OCKRA

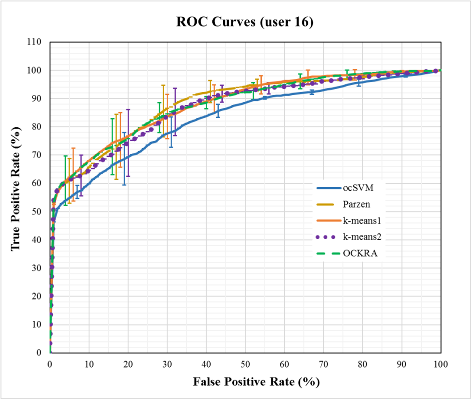
ROC Curves (user 16)
True Positive Rate (%)
False Positive Rate (%)
ocSVM
Parzen
k-means1
k-means2
OCKRA

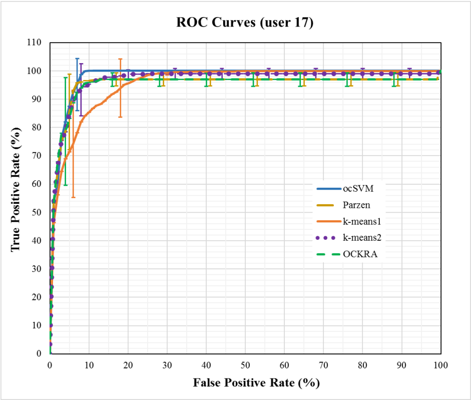
ROC Curves (user 17)
True Positive Rate (%)
False Positive Rate (%)
ocSVM
Parzen
k-means1
k-means2
OCKRA

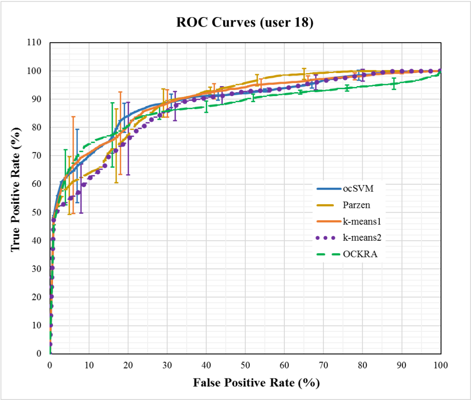
ROC Curves (user 18)
True Positive Rate (%)
False Positive Rate (%)
ocSVM
Parzen
k-means1
k-means2
OCKRA

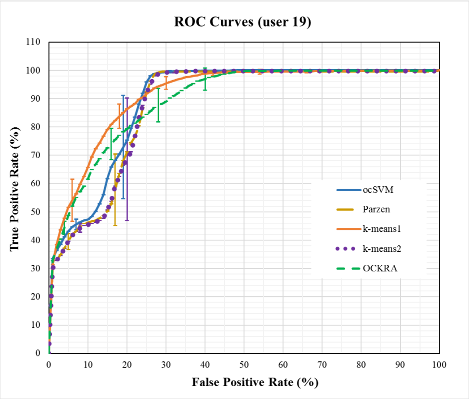
ROC Curves (user 19)
True Positive Rate (%)
False Positive Rate (%)
0
10
20
30
40
50
60
70
80
90
100
110
ocSVM
Parzen
k-means1
k-means2
OCKRA

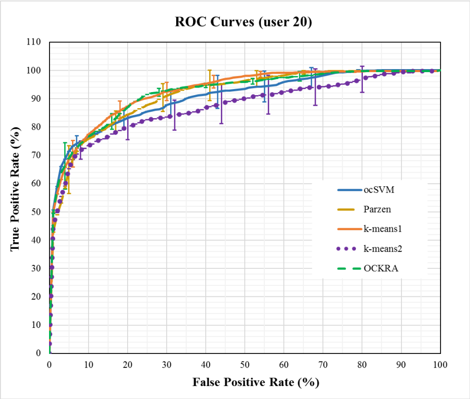
ROC Curves (user 20)
True Positive Rate (%)
False Positive Rate (%)
0
10
20
30
40
50
60
70
80
90
100
110
ocSVM
Parzen
k-means1
k-means2
OCKRA

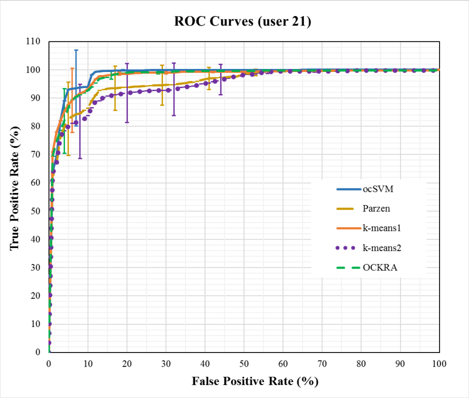
ROC Curves (user 21)
True Positive Rate (%)
False Positive Rate (%)
0
10
20
30
40
50
60
70
80
90
100
110
ocSVM
Parzen
k-means1
k-means2
OCKRA

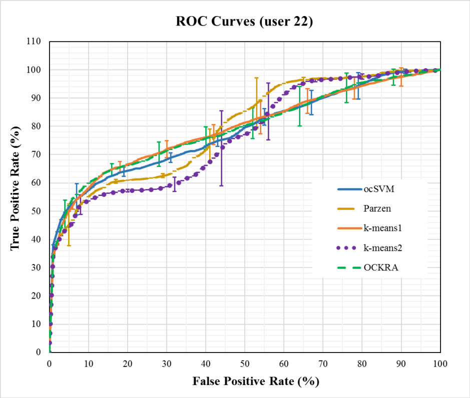
ROC Curves (user 22)
True Positive Rate (%)
False Positive Rate (%)
0
10
20
30
40
50
60
70
80
90
100
110
ocSVM
Parzen
k-means1
k-means2
OCKRA

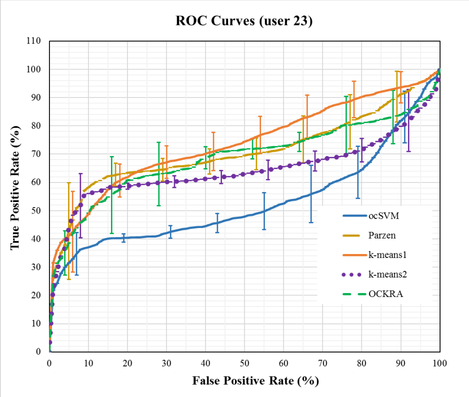
ROC Curves (user 23)
True Positive Rate (%)
False Positive Rate (%)
0
10
20
30
40
50
60
70
80
90
100
110
ocSVM
Parzen
k-means1
k-means2
OCKRA
