# Supplementary figures and images for: Ensemble of One-Class Classifiers for Personal Risk Detection Based on Wearable Sensor Data
Source: Sensors (Basel). 2016 Sep 29;16(10):1619. doi: 10.3390/s16101619 (PMC5087407; doi:10.3390/s16101619)

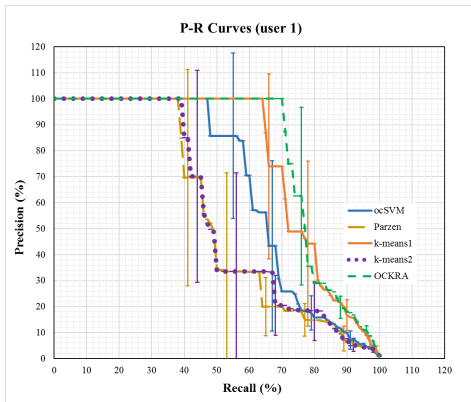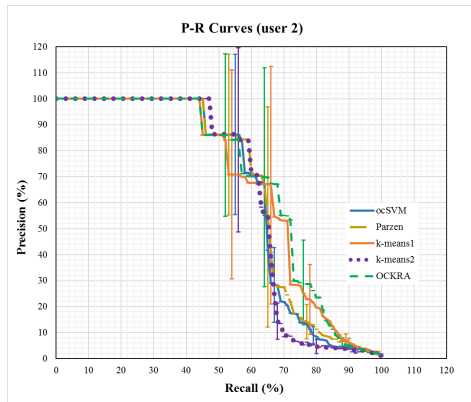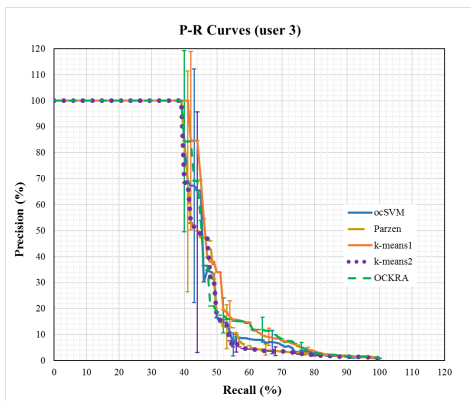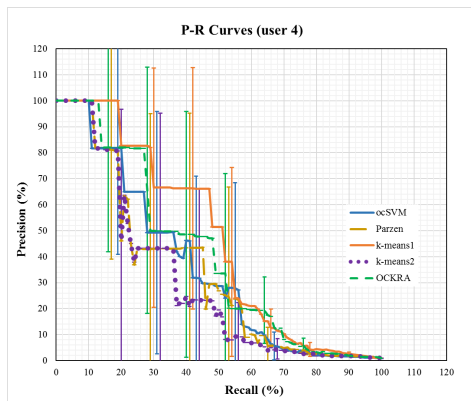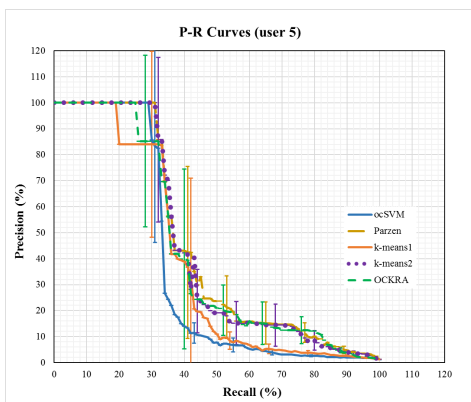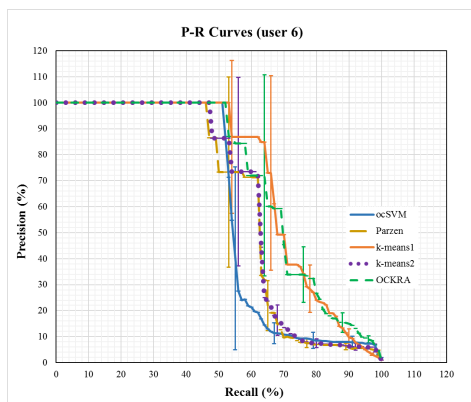

P-R Curves (user 7)

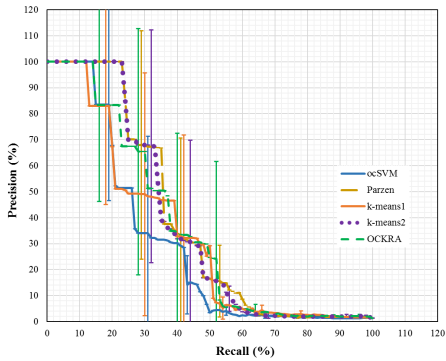

P-R Curves (user 8)

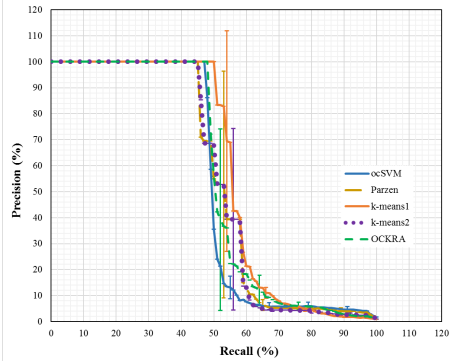

P-R Curves (user 9)

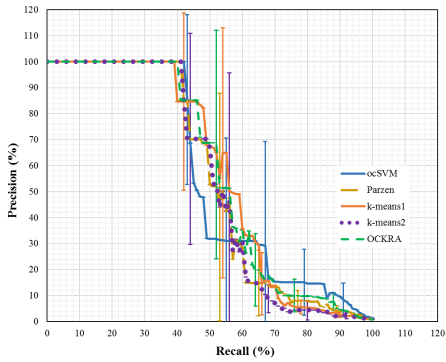

P-R Curves (user 10)

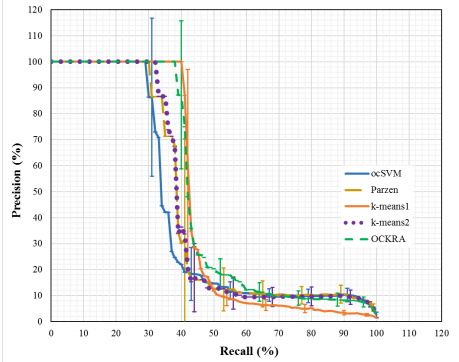

P-R Curves (user 11)

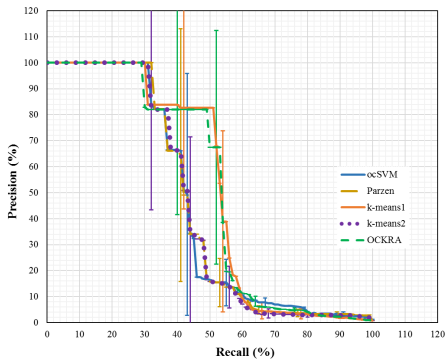

P-R Curves (user 12)

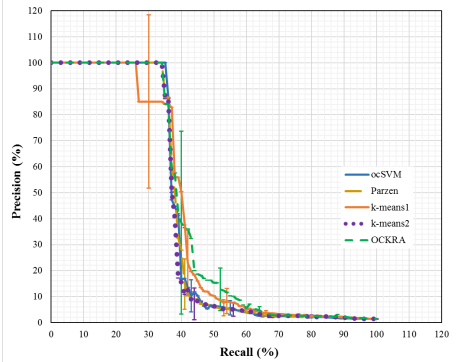

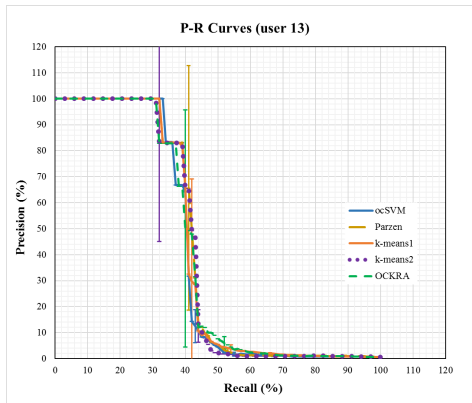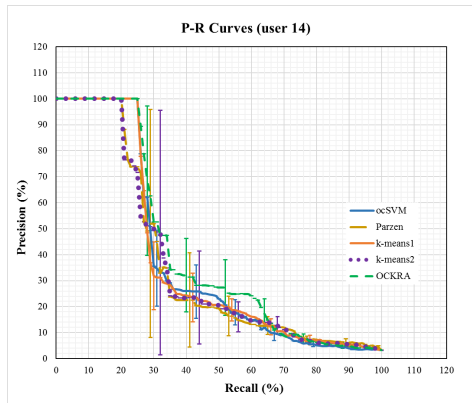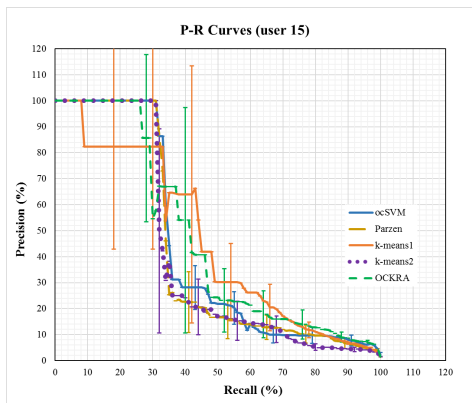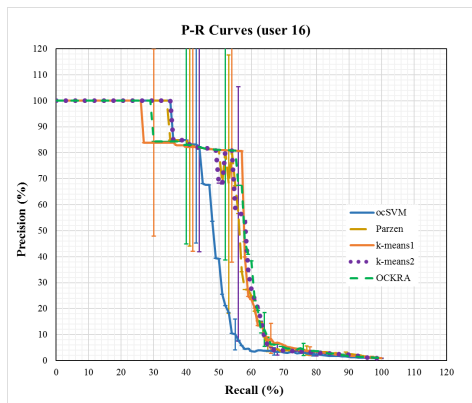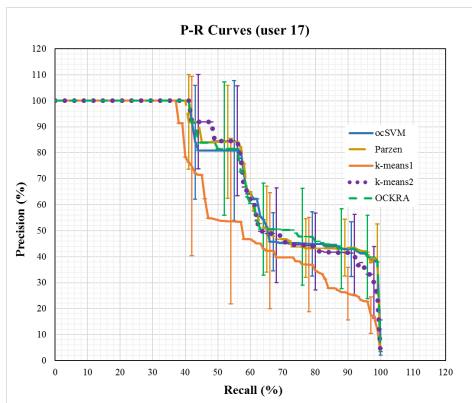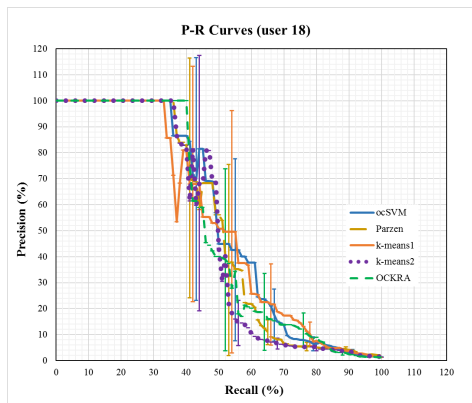

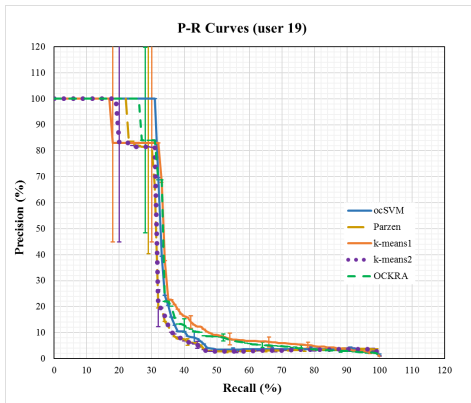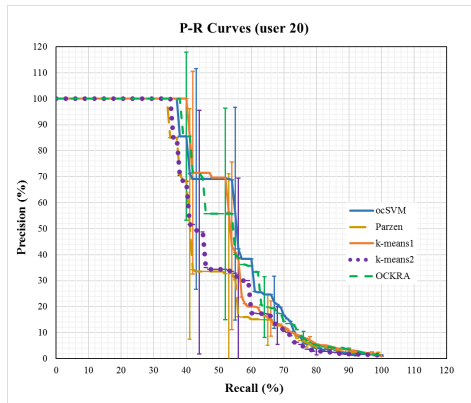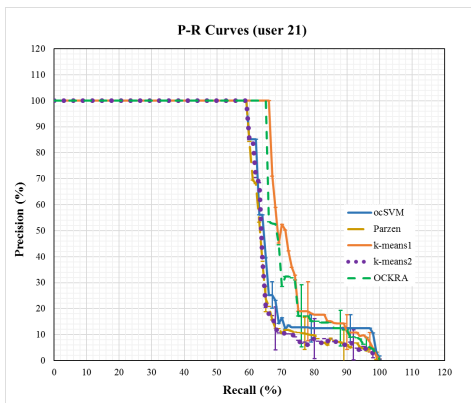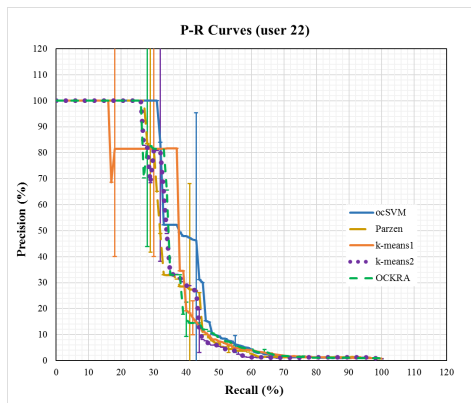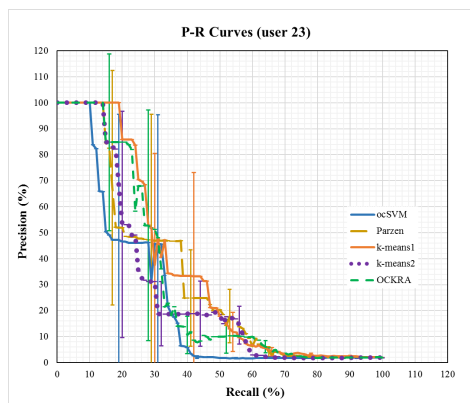

Supplement: Supplementary file 1 [file sensors-16-01619-s001.zip › SupplementaryFiles/Individual P-R Curves.pdf]
